# Mechanisms of synergy of radiotherapy and immunotherapy

**DOI:** 10.1186/1479-5876-13-S1-K5

**Published:** 2015-01-15

**Authors:** Karsten A Pilones, Ryan Emerson, Silvia C Formenti, Harlan S Robbins, Sandra Demaria

**Affiliations:** 1 Department of Pathology NYU School of Medicine, and NYU Cancer Institute, New York, New York, USA; 2 Adaptive Biotechnologies, Seattle, Washigton, USA; 3 Department of Radiation Oncology, NYU School of Medicine, and NYU Cancer Institute, New York, New York, USA; 4 Public Health Sciences Division, Fred Hutchinson Cancer Research, Seattle, Washington , USA

## 

The success of immune checkpoint inhibitors in inducing tumor regression has demonstrated that specific inhibitory pathways are dominant rate-limiting steps in a significant number of patients with melanoma and other advanced cancers. However, in the majority of patients tumor rejection is hindered by multiple immunosuppressive mechanisms present in the tumor microenvironment. Obstacles to immune-mediated tumor control can be present at both the priming and effector phase of the anti-tumor response, and include defective function and activation of antigen-presenting cells, defective T cell recruitment and infiltration of tumors, and defective recognition and killing of cancer cells by T cells. Ionizing radiation therapy (RT) applied locally to a tumor at therapeutic doses has multiple effects that can potentially overcome each of these obstacles, and we have shown that RT is synergistic with immunotherapy. In pre-clinical models RT converted tumors unresponsive to anti-CTLA-4 mAb into responsive ones, achieving rejection of the irradiated tumor and non-irradiated metastases (abscopal effect) and improved survival [1,2]. At the effector phase, RT enhanced recruitment of activated T cells to the tumor by induction of chemokines [3], and enhanced immune synapse formation between CD8 T and tumor cells by induction of NKG2D ligands [4].

To test the hypothesis that successful tumor rejection induced by RT+anti-CTLA-4 requires a significant change in the quantity and quality of tumor-infiltrating lymphocytes (TILs) we performed a comprehensive evaluation of the breadth and depth of the T cell repertoire modulated by treatment using high-throughput sequencing technology. To gain insights into the changes in TILs induced by anti-CTLA-4 treatment in tumor hosts that respond or do not respond to therapy we used our well-characterized 4T1 mouse model in which anti-CTLA-4 treatment is effective only when combined with RT. Results show distinct contributions of RT and anti-CTLA-4 to increasing the number and clonality of TILs, and changes in clonal representation that are unique to the combination. These data suggest that RT effectively releases endogenous tumor antigens that prime anti-tumor T cells, supporting the concept that it can be used as a mean to generate an in situ individualized vaccine. We are currently exploring this hypothesis in clinical trials testing the combination of RT and checkpoint inhibitors.

## References

[B1] Demaria S Kawashima N Yang AM Devitt ML Babb JS Allison JP Formenti SC Immune-mediated inhibition of metastases after treatment with local radiation and CTLA-4 blockade in a mouse model of breast cancer Clin Cancer Res 2005 11 2 728 734 15701862

[B2] Dewan MZ Galloway AE Kawashima N Dewyngaert JK Babb JS Formenti SC Demaria S Fractionated but not single-dose radiotherapy induces an immune-mediated abscopal effect when combined with anti-CTLA-4 antibody Clin Cancer Res 2009 15 17 5379 5388 10.1158/1078-0432.CCR-09-0265 19706802 PMC2746048

[B3] Matsumura S Wang B Kawashima N Braunstein S Badura M Cameron TO Babb JS Schneider RJ Formenti SC Dustin ML Demaria S Radiation-induced CXCL16 release by breast cancer cells attracts effector T cells J Immunol 2008 181 5 3099 3107 10.4049/jimmunol.181.5.3099 18713980 PMC2587101

[B4] Ruocco MG Pilones KA Kawashima N Cammer M Huang J Babb JS Liu M Formenti SC Dustin ML Demaria S Suppressing T cell motility induced by anti-CTLA-4 monotherapy improves antitumor effects J Clin Invest 2012 122 10 3718 3730 10.1172/JCI61931 22945631 PMC3461908

